# Impaired Expression of Focal Adhesion Kinase in Mesenchymal Stromal Cells from Low-Risk Myelodysplastic Syndrome Patients

**DOI:** 10.3389/fonc.2017.00164

**Published:** 2017-08-08

**Authors:** Yuenv Wu, Carmen Mariana Aanei, Sanae Kesr, Tiphanie Picot, Denis Guyotat, Lydia Campos Catafal

**Affiliations:** ^1^Claude Bernard University Lyon 1, Lyon, France; ^2^UMR 5239, Laboratoire de Biologie et Modélisation de la Cellule, Lyon, France; ^3^Laboratoire d’Hématologie, CHU de Saint-Etienne, Saint-Etienne, France; ^4^Département d’Hématologie, Institut de Cancérologie Lucien Neuwirth, Saint-Priest-en-Jarez, France

**Keywords:** myelodysplastic syndromes, mesenchymal stromal cells, focal adhesion kinase, bone marrow microenvironment, ineffective hematopoiesis

## Abstract

The pathogenic role of mesenchymal stromal cells (MSCs) in myelodysplastic syndromes (MDS) development and progression has been investigated by numerous studies, yet, it remains controversial in some aspects (1, 2). In the present study, we found distinct features of MSCs from low-risk (LR)-MDS stromal microenvironment as compared to those from healthy subjects. At the molecular level, focal adhesion kinase, a key tyrosine kinase in control of cell proliferation, survival, and adhesion process, was found profoundly suppressed in expression and activation in LR-MDS MSC. At a functional level, LR-MDS MSCs showed impaired growth and clonogenic capacity, which were independent of cellular senescence and apoptosis. The pro-adipogenic differentiation and attenuated osteogenic capacity along with reduced SDF-1 expression could be involved in creating an unfavorable microenvironment for hematopoiesis. In conclusion, our experiments support the theory that the stromal microenvironment is fundamentally altered in LR-MDS, and these preliminary data offer a new perspective on LR-MDS pathophysiology.

## Introduction

Myelodysplastic syndromes (MDS) are heterogeneous clonal hematopoietic stem cells disorders, characterized by ineffective hematopoiesis with varying degrees of peripheral cytopenia and increased risk of acute myeloid leukemia (AML) transformation (3). Evidence suggesting the involvement of bone marrow microenvironment in ineffective hematopoiesis besides intrinsic HSPC abnormalities increased in recent years (4, 5).

Mesenchymal stromal cells (MSCs), which represent one of the main cellular components in bone marrow microenvironment and actively participate in hematopoiesis regulation, are found somehow to be less capable of fulfilling its duties in MDS, whether based on genomic alteration (4, 6, 7) or not (2). Despite being intensely studied, the definition of pathogenic roles of MSC in MDS pathophysiology is far from complete. Therefore, new perspectives are needed in advancing our knowledge of underlying abnormalities in MDS microenvironment.

Focal adhesion kinase (FAK) is a cytoplasmic tyrosine kinase. Once activated, it can initiate a cascade of signaling events through PI3K-Akt, MAPK, promoting cell survival, growth, mobility, and adhesion (8). It is well known that FAK is an aggressive marker in several advanced-stage solid cancers (9, 10) and hematologic malignancies (11, 12).

Focal adhesion kinase overexpression and overactivation bestow a proliferation and survival advantage upon cancer cells (9–12). Interestingly, previous work from our team showed that increased expression of FAK and its strong association with HSP90α/β in cellular nuclear complexes were fundamental features of MSCs in refractory anemia with excess blasts (RAEB; a high-risk subtype of MDS), which was correlated with a higher proliferation capacity and decreased hematopoiesis support (13). MDS are heterogeneous groups of diseases with distinct clinical features and prognosis. Consequently, it is important to determine whether aberrant FAK expression/activation is also involved in the abnormal microenvironment of these MDS subtypes.

In the present study, we investigated whether the microenvironment of LR-MDS is responsible for the ineffective hematopoiesis, the major trait at this stage of the disease. We are interested to evaluate the hematopoiesis-supporting capacity of LR-MDS MSCs and to determine the possible involvement FAK deficiency in inducing MSC abnormalities.

## Materials and Methods

### Patients and Samples

Bone marrow aspirates were collected from 19 untreated, *de novo* MDS subjects with a mean age of 80 ± 7 years (range: 64–89) and 9 healthy subjects with mean age of 60 ± 17 years (range: 39–88) as healthy controls (HC). MDS “low-risk” (LR) [International Prognostic Scoring System (IPSS), low and intermediate-1] patients were included in this study. Bone marrow specimens were obtained with a signed informed consent, and the study was approved by our local institutional review board.

Table 1 shows the distribution of the cases according to the 2008 WHO classification and IPSS.

**Table 1:** Myelodysplastic syndrome patients diagnostic and risk stratification.

Diagnosis	No.	%
MDS-U	1	5
MDS 5q-	2	11
MDS RA	4	21
MDS RCMD	11	58
MDS RAEB-I	1	5
IPSS		
Low	9	47
Intermediate-1	10	53

*MDS 5q-, myelodysplastic syndrome with isolated del5q; MDS-U, myelodysplastic syndrome-unclassified; RAEB, refractory anemia with excess blasts; RCMD, refractory cytopenia with multilineage dysplasia; IPSS, international prognostic scoring system*.

The “Comité de Protection des Personnes” (Independent Ethics Committee) Sud-Est 1 from the University Hospital of Saint-Etienne, France, has reviewed and given ethical approval for the project entitled “Evaluation of the intercellular cross talk between (MSCs) and hematopoietic stem progenitor cells in MDS: role of FAK and of FAK regulators?”

### MSC Isolation and Expansion

Bone marrow mononuclear cells were isolated by density centrifugation; then the cells from LR-MDS and control group were cultured in MesenCult^®^ MSC basal medium (Stem Cell Technology, Canada) containing 1% penicillin/streptomycin/l-Glutamine (Gibco^®^, USA) at a temperature of 37°C in a humidified incubator with 5% CO_2_ atmosphere. After 24 h incubation, the culture medium was replaced, and non-adherent cells were removed. When 80% confluence was reached, the MSCs were detached with 0.25% trypsin-EDTA. All experiments were carried out using MSC derived from passages 1–3.

### MSC Proliferation Assay

Mesenchymal stromal cell proliferation was indicated by the quantity of viable cells after 7 and 14 days in culture using the MST assay (CellTiter 96^®^AQueous, Promega, France). The value (determined by optical density) of each sample was compared to the basal MST absorbance readings, as shown previously (14). MSCs were seeded in three replicative wells on day 0 at equal cell densities (1 × 10^3^ MSCs per well). The absorbance reading on day 1 was used as the baseline reading.

### MSC Differentiation Assay

Adipogenic and osteogenic differentiation of MSC from LR-MDS and control group were induced by AdipoDiff/OsteoDiff medium (Miltenyi Biotec, Germany). MSC-adipogenic differentiation has been performed during 21 days. Then cells were fixed and stained with Oil Red O (Sigma-Aldrich, USA) to detect lipid accumulation. Cells with lipid droplets (adipocytes) were counted under a light microscope. After 10 days of osteogenic induction, the mineralization capacity of MSCs was detected by staining with Alizarin Red S (Sigma-Aldrich, USA). For the purpose of quantification, the osteogenic differentiation was graded according to microscopic analysis of staining intensity as follows: 0 = absent; 1 = 20%; 2 = 40%; 3 = 60%; 4 = 80%; and 5 = 100%. Duplicate experiments have been made for each sample.

### Cellular Senescence Analysis

SA-galactosidase activity was performed in order to determine cell senescence in LR-MDS and HC-MSCs using the Cellular Senescence Detection Kit (Cell Signaling Technology, USA). SA-galactosidase-positive cells (stained in blue color) were counted by light microscopy. Each sample was treated in duplicate.

### Flow Cytometry

To fulfill the criteria of the International Society for Cellular Therapy, MSCs were analyzed for the expression of CD34, CD45, CD73, CD90, CD105, and CD44 (fluorochrome-conjugated monoclonal antibodies from BD Bioscience) by flow cytometry (FACSCanto II Becton Dickinson, BD Bioscience, USA). Cellular apoptosis was evaluated by Annexin V and 7AAD staining, detected by FACSCanto II Becton Dickinson (BD Bioscience, USA). Unstained MSCs were used as negative controls to assess background fluorescence. Cell analysis was performed using FlowJo software (TreeStar Inc., USA).

### Quantitative Real-time PCR

Total RNA was extracted from LR-MDS and HC-MSCs when MSC achieved 80% confluence. Extraction was made with TRIZOL (Invitrogen, USA) followed by reverse transcription for cDNA synthesis. Gene expression of CDKN1A (primers: Fw: 5′-GAGGCCGGGATGAGTTGGGAGGAG -3′; Rv: 5′-CAGCCGGCGTTTGGAGTGGTAGAA -3′), PTK2 (primers: Fw: 5′-GCTTACCTTGACCCCAACTTG-3′; Rv: 5′-ACGTTCCATACCAGTACCCAG-3′), and SDF-1 (primers: Fw: 5′-ATGAACGCCAAGGTCGTG-3′; Rv: 5′-ACATGGCTTTCGAAGAATCG-3′) was detected by the SYBR Green method in 7900HT Fast Real-time PCR System (Applied Biosystems, USA). Relative gene expression was calculated using the 2^−ΔΔct^ method after normalization to the reference gene GAPDH (primers: Fw: 5′-AATCCCATCACCATCTTCCAGG-3′; Rv: 5′-AGAGGCAGGGATGATGTTCTGG-3′).

### Western Blot Analysis

The protein concentration of MSC lysates was quantified by the BCA protein assay kit (Pierce, USA). Equal amounts of protein from each sample were loaded and run on PAGE 4–15% gels (Bio-Rad, USA), and then they were transferred onto the Hybond ECL membrane (GE Healthcare, UK). Blots were blocked with 5% non-fat milk in TBS-T buffer for 1 h and then incubated for 2 h with anti-FAK (Cell Signaling Technology, USA), anti-p-FAK(Y397) (Thermo Fisher, USA), and anti-actin antibody (BD Bioscience, USA). After three washes with TBS-T buffer, HRP-conjugated anti-mouse and anti-rabbit antibody (DAKO, Denmark), a 1-h staining with secondary antibodies has been performed. Thereafter, three washes with TBS-T buffer were performed to leave out the excess of antibodies. Finally, the bands were visualized by using Clarity Western ECL Substrate (Bio-Rad, USA) in Hyperfilms (Amersham, UK). The densitometry evaluation and the comparison of bands on the same membrane were performed in Adobe Photoshop CC version 2017.0.020161012.r.53X64.

### Statistical Analysis

Data are presented as mean and SEM. Comparisons of the data of each group were performed by two-tailed *t-*test using Prism 5.0c (GraphPad Software, USA). Statistical significance was set as *p ≤ 0.05; **p ≤ 0.005; ***p ≤ 0.001.

## Results

### Altered Morphology and Colony Structure during LR-MDS-MSCs Expansion

**Figure 1:**
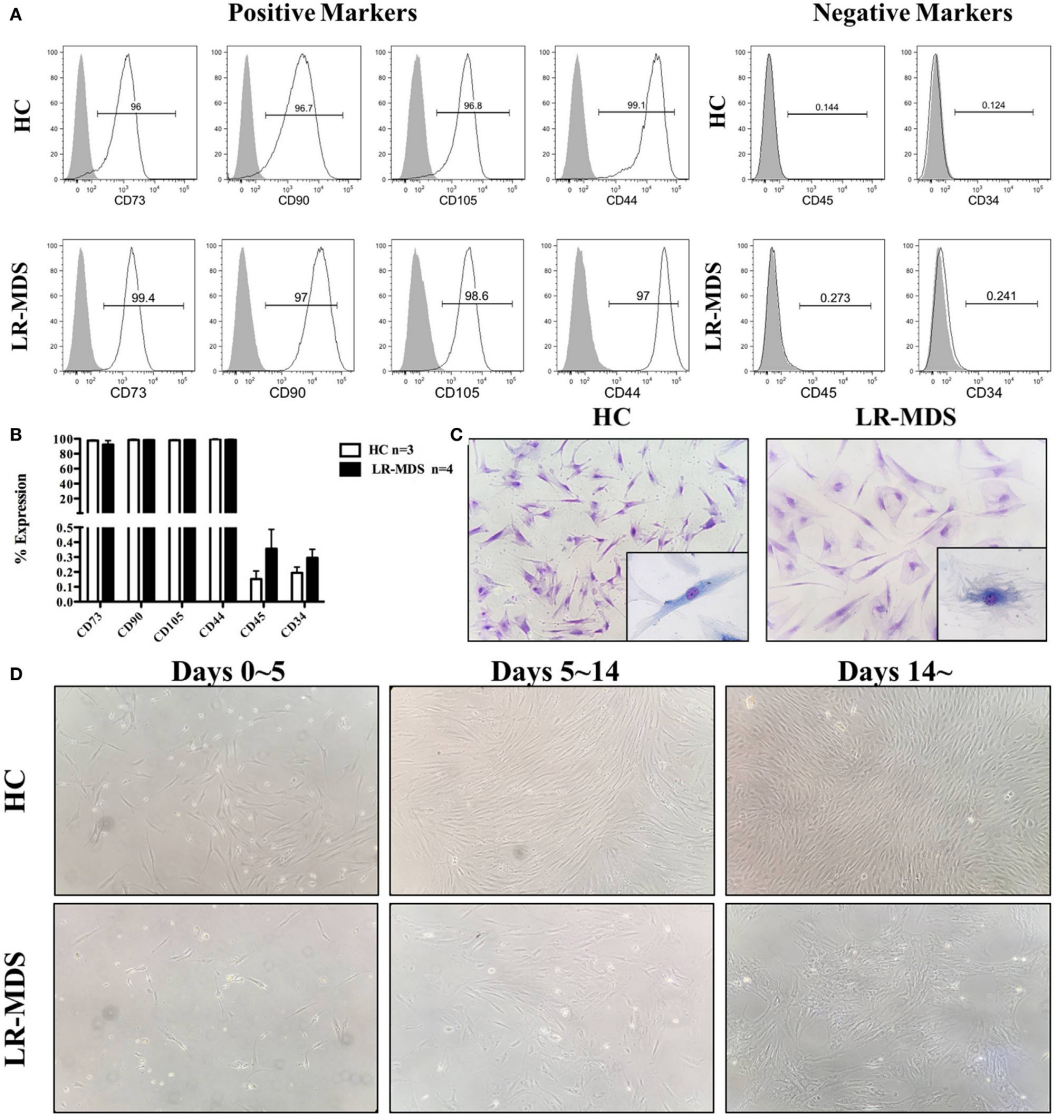
Immunophenotype and morphology of low-risk myelodysplastic syndromes (LR-MDS) and healthy controls (HC)-mesenchymal stromal cells (MSCs). Percentages of cells positive for CD34, CD45, CD73, CD90, CD105, and CD44 were determined in LR-MDS and HC-MSCs by flow cytometric analysis **(A)**. Representative images depict morphology of LR-MDS and HC-MSCs **(B)**. Representative examples of colony architecture in primary cultures of LR-MDS **(C)** and HC-MSCs **(D)**.

The expression of MSC-specific markers was performed by cytometry in order to determine the purity of MSC from LR-MDS and HC. Both groups of cells displayed typical MSC markers as they were positive for CD73, CD90, and CD105 (>95% of cells) and lacked the hematopoietic markers of CD45 and CD34 (<1% of cells) (Figures 1A,B). MSCs morphology was examined by light microscopy with or without 0.1% toluidine blue staining. As showed in Figure 1C, HC-MSCs displayed a characteristic fibroblast-like appearance, whereas LR-MDS MSCs were generally larger, appearing flatten, and irregular in shape. Moreover, we observed a disrupted colony architecture formed by LR-MDS-MSC, which consisted of loosely connected cells that expanded in a disorganized way (Figure 1D). HC-MSCs, on the other hand, produced smooth confluence (Figure 1D).

### Intrinsic Growth Impairment in LR-MDS-MSCs

In addition to the aberrant colony architecture, the proliferative capacity of MSCs of LR-MDS was lower than in normal control group.

**Figure 2:**
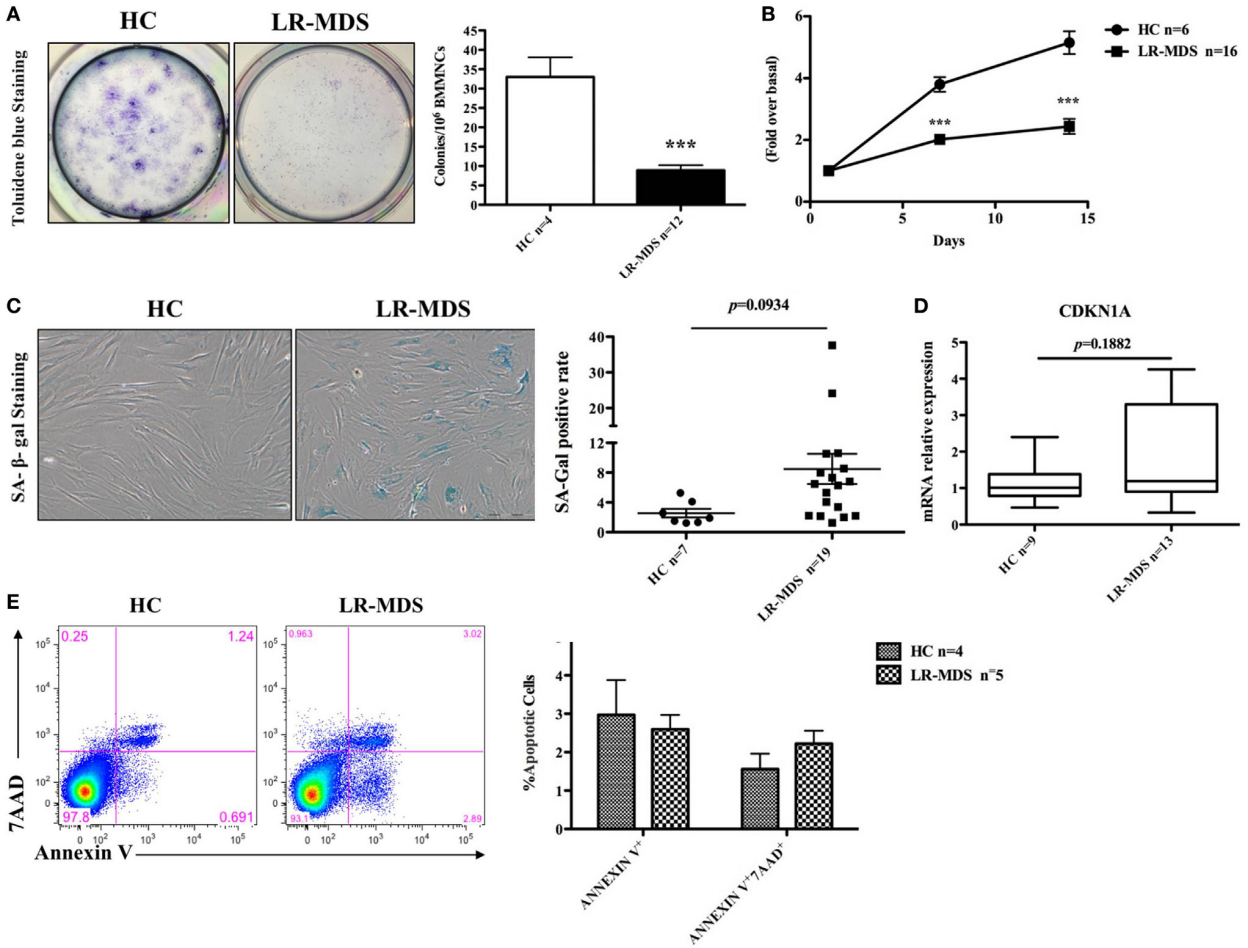
Clonogenic capacity and cell growth of low-risk myelodysplastic syndromes (LR-MDS) and healthy controls (HC)-mesenchymal stromal cells (MSCs). CFU clonogenic capacity evaluation **(A)**. MSCs from LR-MDS and HC were seeded at the same density at day 0. MST assay was performed at day 1 (baseline setting), day 7, and day 14 **(B)**. SA-galactosidase-positive cells (shown in blue color) were numerated per 200 consecutively counted MSCs **(C)**. Gene expression of CDKN1A was detected by real-time PCR and normalized using the relative quantity of GAPDH as an endogenous control **(D)**. Evaluation of apoptosis in HC and LR-MDS MSCs was performed by flow cytometry. **(E)** Depicts the percentages of the early apoptotic cells (Annexin V^+^7AAD^−^) and late apoptotic phase (Annexin V^+^7AAD^+^) in HC-MSCs and LR-MDS MSCs.

The growth kinetics of MSCs from HC and LR-MDS have been evaluated in day 1 and day 14 of culture using MST assay. As depicted in Figure 2B, the OD value (indicator of the quantity of cells alive) continues to rise significantly faster in healthy control groups compared with the LR-MDS group (day 7: HC mean ± SEM: 3.8 ± 0.2; LR-MDS mean ± SEM: 2 ± 0.2; day14: HC mean ± SEM: 5.2 ± 0.4; LR-MDS mean ± SEM: 2.4 ± 0.2). Unlike the continued robust proliferation of HC-MSCs within 14 days (HC day 7 vs HC day 14, *p* = 0.012), LR-MDS MSCs remarkably slowed down in expansion soon after day 7 (LR-MDS day 7 vs LR-MDS day 14, *p* = 0.168). Presumably, the proliferation limits were about to be reached if the culture continued, which means that, in MDS cultures, the number of cells counted in day 14 was very similar to that obtained in day 7; the capacity of proliferation is limited in time. We may keep cells in cultures, but they are not able to evolve in time.

Contradicting some reports (4, 14), our results revealed that although LR-MDS MSCs displayed a tendency toward cellular senescence, their senescence level, determined by both the percentage of SA-galactosidase-positive cells (HC mean ± SEM: 2.6 ± 0.6; LR-MDS mean ± SEM: 8.5 ± 2, Figure 2C) and gene expression level of senescence-associated molecule p21 (encoded by CDKN1A) (HC mean ± SEM: 1.1 ± 0.2; LR-MDS mean ± SEM: 1.8 ± 0.4, Figure 2D), was not statistically significant when compared to HC-MSCs. Moreover, the impaired growth of LR-MDS MSCs was not attributed to the excess apoptosis as shown by Annexin V and 7AAD staining. LR-MDS MSCs did not reveal significant differences in apoptosis levels between LR-MDS MSCs compared to HC-MSCs (Annexin V^+^: HC mean ± SEM: 3 ± 0.9; LR-MDS mean ± SEM: 2.6 ± 0.4, *p* = 0.689; Annexin V^+^7AAD^+^: HC mean ± SEM: 1.6 ± 0.4; LR-MDS mean ± SEM: 2.2 ± 0.3, *p* = 0.241, Figure 2E). Collectively, the LR-MDS MSCs were intrinsically pathological, presenting suppressed and disorganized proliferation that was not related to senescence nor apoptosis. Our previously work showed that the proliferation of MSCs selected from MDS correlates substantially with the decline of CD44 and CD49e expression on their surface (14). Consequently, we believe that the mechanism responsible for abnormal proliferative capacity is an adhesion-dependent process.

### LR-MDS-MSCs Exhibit a Higher Propensity toward Adipogenic Differentiation, Which Was Indirectly Correlated with a Reduced HSPC Clonogenic Potential

**Figure 3:**
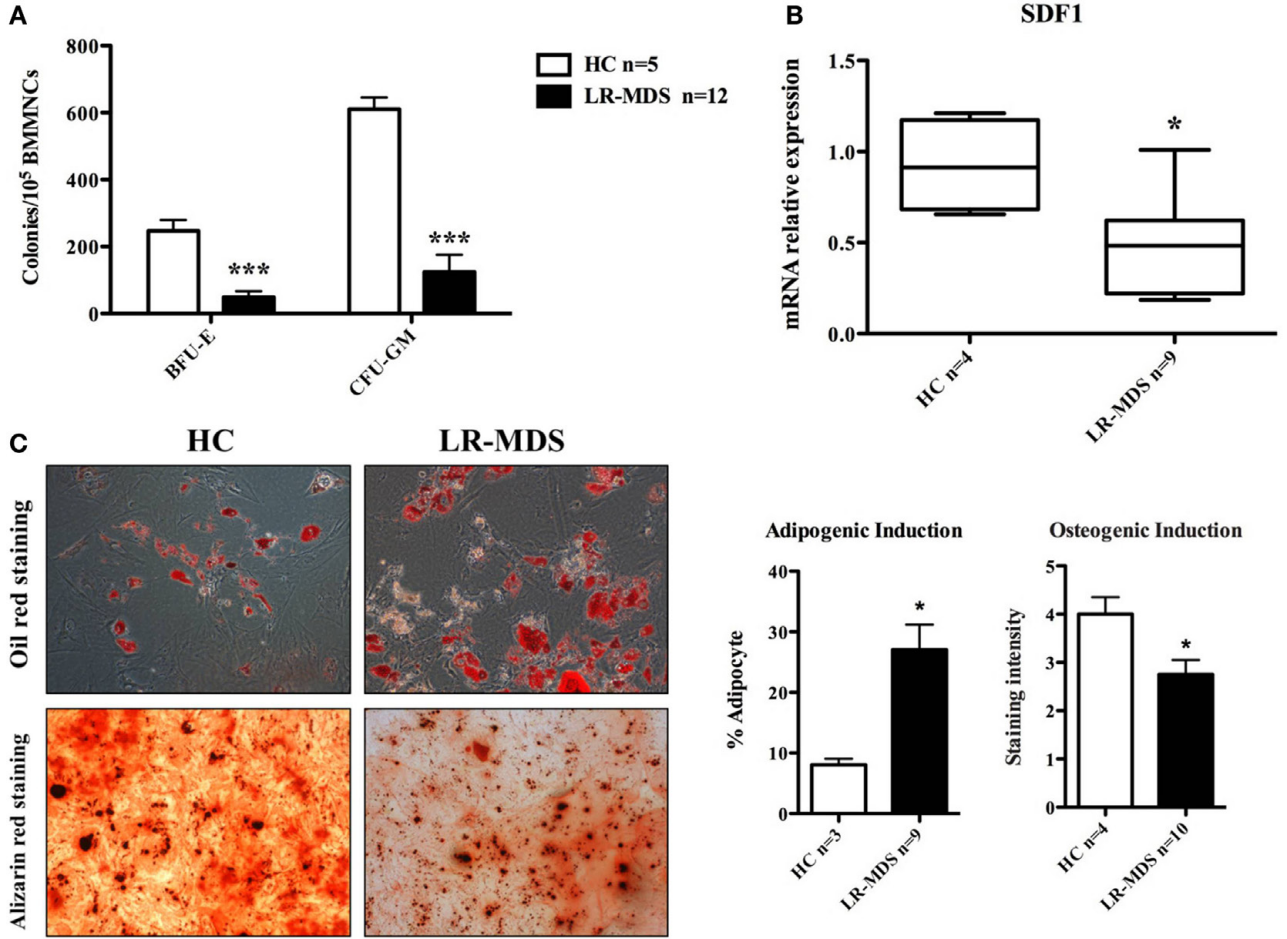
Hematopoiesis-supporting potential of low-risk myelodysplastic syndromes (LR-MDS) and healthy controls (HC)-mesenchymal stromal cells (MSCs). BFU-E, CFU-G, and CFU-M isolated from healthy donors and LR-MDS were counted under a light microscope **(A)**. Evaluation of mRNA expression of SDF-1 in LR-MDS MSC compared to HC **(B)**. LR-MDS and HC-MSCs were cultured in adipogenic and osteogenic induction medium for 21 and 10 days, respectively, according to the protocol of the manufacturer. Then, the differentiation results were quantified by chemical staining **(C)**. All experiments were performed in duplicate.

Ineffective hematopoiesis is a key feature of LR-MDS, as shown herein in CFU assays (Figure 3A). Compared to the HC, the HSPC from LR-MDS were significantly less capable of generating erythroid (HC mean ± SEM: 262 ± 37.3; LR-MDS mean ± SEM: 33.1 ± 15.6) and granulocytic and macrophage colonies (HC mean ± SEM: 624 ± 41.4; LR-MDS mean ± SEM: 125.1 ± 70.9). Concomitantly, the MSC counterpart showed differentiation abnormalities in LR-MDS patients with HSPC low clonogenic capacity as they were increased in adipogenesis (HC mean ± SEM: 8.1 ± 1; LR-MDS mean ± SEM: 27.1 ± 4.1, *p* = 0.0321) and diminished in osteogenesis (HC mean ± SEM: 4 ± 0.4; LR-MDS mean ± SEM: 2.8 ± 0.3, *p* = 0.0361) (Figure 3C). Given that the osteoblasts are key regulators in hematopoietic niche (5), the deregulated program of LR-MDS MSCs differentiation could negatively influence the regulation of hematopoiesis. In addition, the gene expression level of SDF1 (a molecule involved in HSPC localization in the niche) was significantly downregulated in LR-MDS MSC (HC mean ± SEM: 0.92 ± 0.1; LR-MDS mean ± SEM: 0.47 ± 0.1, *p* = 0.017, Figure 3B). Taken together, the deficiency in proliferation, osteogenesis, and SDF-1 expression made LR-MDS MSCs less capable of supporting normal hematopoiesis.

### Downregulation and Decreased Activation of FAK in LR-MDS-MSCs

**Figure 4:**
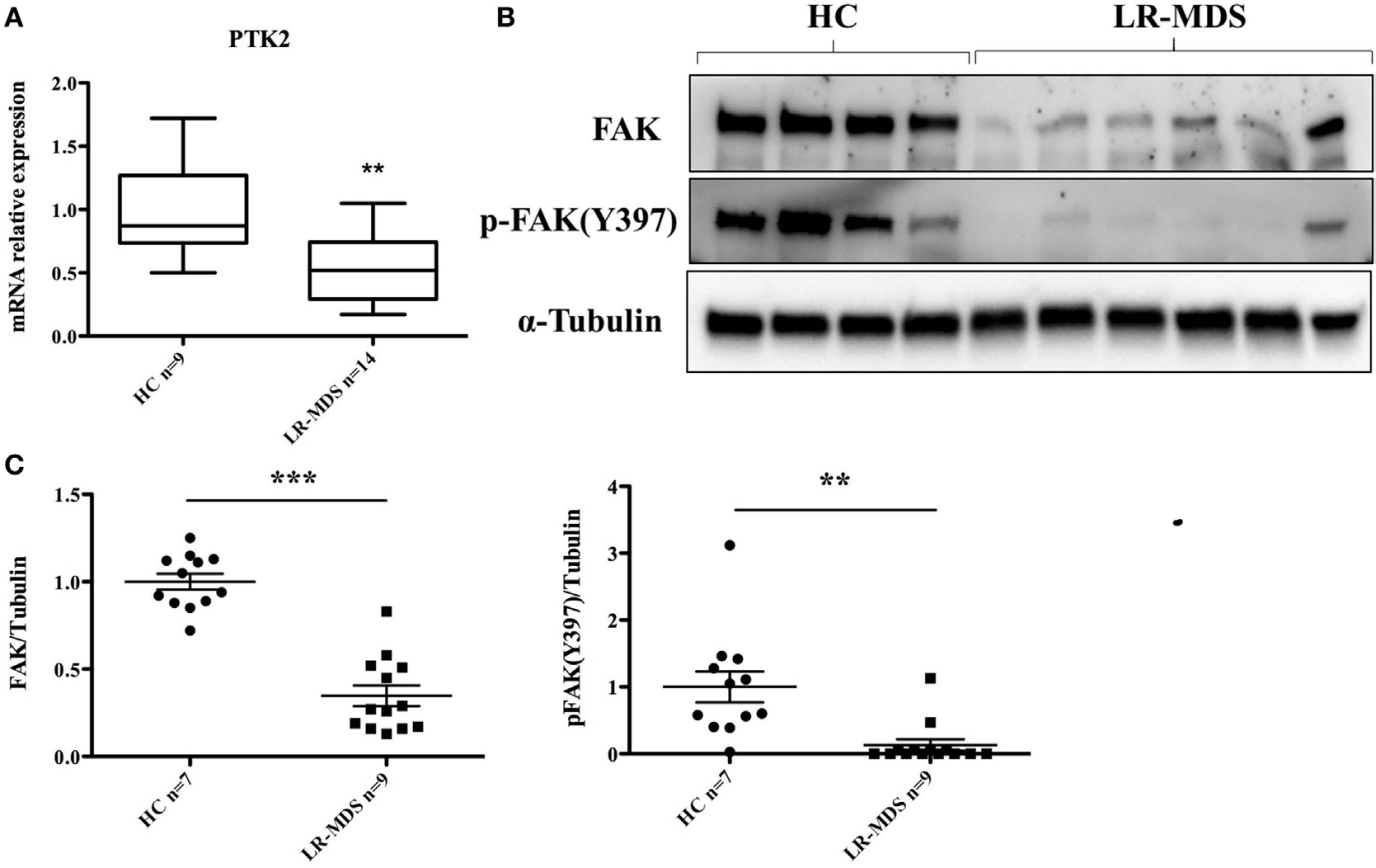
Focal adhesion kinase (FAK) expression and p-FAK (Y397) phosphorylation in low-risk myelodysplastic syndromes (LR-MDS) and healthy controls (HC)-mesenchymal stromal cells. Real-time PCR detection of PTK2 gene expression **(A)**. Protein FAK expression and activation were evaluated by western blot of 4 HC and 6 LR-MDS samples **(B)**. Relative quantification of western blot was carried out by densitometry evaluation of bands on the same membrane; each sample was compared to the mean value of HC **(C)**.

The putative molecular abnormalities were evaluated in LR-MDS MSCs, with special interest for FAK that was previously demonstrated to have been involved in hematopoiesis deregulation in the microenvironment of high-risk MDS (13). We were interested to find whether FAK might be responsible for the abnormal functional capacities of LR-MDS MSCs, including reduced cellular connection during colony formation (Figure 1D), diminished clonogenicity (Figure 2A), impaired proliferation (Figure 2B), and reduced osteogenesis (Figure 3C) compared to HC-MSCs. A significant decrease of FAK was observed in LR-MDS MSCs, both at the gene level (HC mean ± SEM: 1.1 ± 0.1; LR-MDS mean ± SEM: 0.5 ± 0.1, *p* = 0.0003, Figure 4A) and at the protein level (HC mean ± SEM: 1.0 ± 0.1; LR-MDS mean ± SEM: 0.35 ± 0.1, *p* < 0.0001, Figures 4B,C), along with a notable suppression in its activation (evaluated as its ability for auto-phosphorylation at site Y397) (HC mean ± SEM: 1 ± 0.2; LR-MDS mean ± SEM: 0.1 ± 0.1, *p* = 0.0038, Figures 4B,C).

## Discussion

Mesenchymal stromal cells and their progenies in the bone marrow microenvironment actively regulate hematopoiesis. Their contribution to ineffective hematopoiesis in MDS has been recently demonstrated in several seminal studies (4–6, 13–15). In line with other studies, our data support the fact that LR-MDS MSCs are intrinsically pathologic, having altered phenotypes and functions. Notably, these abnormal MSCs from LR-MDS are characterized by a remarkable deficit in adhesion molecule FAK that was downregulated and hypoactivated in this setting (Figures 4A–C).

Focal adhesion kinase regulates a signaling network that orchestrates a wide range of cellular processes including survival, proliferation, differentiation, mobility, and adhesion (8, 16, 17). Overexpression and overactivation of FAK are associated with tumor aggressiveness (9). In recent years, research has been focusing on the role of FAK in tumor microenvironment. Stokes et al. showed, in pancreatic ductal adenocarcinoma, that FAK inhibition targets both the tumor and the surrounding stromal cells exerting significantly greater antitumor effect than targeting tumor cell alone (18). In a 3D experiment, tumor growth and invasion was blocked when tumor cells were cocultured with cancer-associated fibroblasts (FAK^−/−^), which inhibit their proliferation and motility (15). FAK inhibition can also abrogate niche-formation capacity of MSC when the MSCs have been cocultured with different solid tumor cell lines (19). In normal tissue microenvironment, FAK is shown to regulate MSCs differentiation (16, 17) and coordinate the interaction between endothelial cells and lymphocytes (19).

Our previous work illustrated a distinct role of MSCs bearing an overactivated FAK in abnormal, tumor-supporting RAEB microenvironment (13). This finding encouraged us to evaluate whether FAK is abnormally expressed and contributes to the abnormal microenvironment in LR-MDS. Based on other evidence derived from studies of solid tumor microenvironment (15, 17–22), it is reasonable to speculate the contribution of FAK and p-FAK(Y379) to the disease progression toward HR-MDS/AML. A putative explanation is that MSCs, bearing an abnormal expression of FAK either spontaneously or secondary to the reprogramming and selection by the blasts cells, may promote MDS evolution and even AML transformation. Unlike HR-MDS, LR-MDS patients usually present limited number of blasts in bone marrow; and the improvement of hematopoiesis largely depends on proper functioning of the microenvironment. Therefore, our interest was to focus on detection of the underlying abnormalities of the microenvironment in LR-MDS, with particular interest in MSCs.

A significantly reduced expansion potential of LR-MDS MSCs was detected compared to HC-MSCs (Figures 2A,B). In addition, the MSCs selected from LR-MDS have generated limited small colonies (Figure 2A), where MSCs were loosely connected and expanded in a disorganized manner (Figure 1D). By contrast, the disrupted colony pattern was not seen in HC-MSCs. The altered morphology and colony architecture disruption were previously mentioned in some reports, but without offering a putative explanation to this abnormality (4, 21). Our preliminary data showed an augmented FAK expression and activation in HR-MDS, and AML MSCs were associated with a tendency to form intensely compact colonies (data not shown). The altered MSC–MSC interaction may contribute to the abnormal MSC–HSPC interaction, but this hypothesis requires further investigation.

The abnormal proliferation of MDS MSCs is a controversial topic: a part of the authors claiming a significant altered growth kinetics (4, 23), others demonstrating a normal growth pattern, very similar to HC (2). Our results support the first observation. The LR-MDS MSCs were much less capable of proliferating as fast as HC-MSCs, and their cultures were not sustainable in the long term (Figure 2B). This reduced proliferative capacity was independent of cellular senescence (Figures 2C,D), which contradicted a part of other reports (4, 21). An increased tendency toward senescence in LR-MDS MSCs has been observed in our culture systems, but the differences with normal counterparts did not reach statistical significance. This discrepancy may be related to the differences in experimental methodology and patient selection. We presume that the downregulation and inactivation of FAK in LR-MDS may drive the abnormal MSC proliferation.

Moreover, the LR-MDS MSCs tendency toward an imbalanced differentiation (Figure 3C) could influence the normal hematopoiesis in the bone marrow. It had been demonstrated that FAK is actively involved in both adipogenesis and osteogenesis processes, mainly through BMP-Smad signaling (16, 17). Presumably, other molecules, upstream or downstream FAK, might be involved in the MDS pathogenic process besides FAK.

Collectively, our data suggest that the LR-MDS MSCs are morpho-phenotypically and functionally different compared to HC-MSCs, showing a limited and aberrant expansion pattern and a differentiation program skewed toward adipogenesis. This abnormal differentiation ability correlates with the significant FAK downregulation and hypophosphorylation. Whether FAK defect is directly responsible for these abnormalities in LR-MDS MSCs and how it influences MSC–HSCP interaction leading to ineffective hematopoiesis are currently under investigation.

## Author Contributions

All authors listed have made a substantial, direct and intellectual contribution to the work, and approved it for publication.

## Conflict of Interest Statement

The authors declare that the research was conducted in the absence of any commercial or financial relationships that could be construed as a potential conflict of interest.
